# Peripherally inserted central catheter-related bloodstream infection due to *Tsukamurella pulmonis*: a case report and literature review

**DOI:** 10.1186/s12879-017-2796-8

**Published:** 2017-10-11

**Authors:** Jun Suzuki, Teppei Sasahara, Masaki Toshima, Yuji Morisawa

**Affiliations:** 1 Division of Infectious Diseases, Jichi Medical University Hospital, 3311-1 Yakushiji, Shimotsuke, Tochigi, 329-0498 Japan; 2 Department of Infection and Immunity, School of Medicine, Jichi Medical University, 3311-1 Yakushiji, Shimotsuke, Tochigi, 329-0498 Japan

**Keywords:** *Tsukamurella pulmonis*, Acquired immunodeficiency syndrome (AIDS), Diffuse large B cell lymphoma (DLBCL), Peripherally inserted central catheters (PICCs), Catheter-line associated bloodstream infection (CLABSI)

## Abstract

**Background:**

*Tsukamurella pulmonis* is an aerobic gram-positive and rod-shaped organism that causes central catheter-related bloodstream infections in immunocompromised hosts. However, peripherally inserted central catheter (PICC)-related bloodstream infections due to this organism have not been reported.

**Case presentation:**

We describe a case of a 48-year-old man with acquired immunodeficiency syndrome and diffuse large B cell lymphoma who received five courses of chemotherapy including rituximab, cyclophosphamide, doxorubicin hydrochloride, vincristine, and prednisone via a PICC. Five days after the last chemotherapy course, he presented with a high fever and shaking chills. His absolute neutrophil count was 4200/μL. Cultures obtained from blood and PICC culture revealed *T. pulmonis*. The colony count of *T. pulmonis* grown from PICC culture was 10^3^ colony-forming units. Therefore, he was diagnosed with *T. pulmonis* bacteremia resulting from PICC-related bloodstream infection. The patient’s condition improved and he became afebrile within 48 h after intravenous administration of cefozopran hydrochloride, which is a fourth generation cephalosporin.

**Conclusions:**

PICCs can be associated with *T. pulmonis* bacteremia, and fourth generation cephalosporins may be effective treatment.

## Background

*Tsukamurella* species are aerobic gram-positive and rod-shaped organisms with a specific cell wall chemistry that separates them from aerobic actinomycetes [1]. Although infections caused by *Tsukamurella* species are rare, these species can cause nosocomial infections in immunocompromised hosts [2]. A report from 1996 described isolation of *Tsukamurella pulmonis* from the sputum of a 92-year-old woman with lung mycobacterium infection [3]. *T. pulmonis* infections are associated with hematological and immunosuppressed patients, and the reported observations of the infection include conjunctivitis [4], keratitis [5], pneumonia [6], and central catheter-related bloodstream infections [7].

Peripherally inserted central catheters (PICCs) have been popular because of their safety, easy insertion, and lower rates of infection [8–10]. Although the use of central venous catheters (CVCs) and Hickman catheters is a reported cause of catheter-line associated bloodstream infection (CLABSI) due to *T. pulmonis* [7], PICC-related bloodstream infections with this organism have not been reported.

Here, we present the case of 48-year-old patient with acquired immunodeficiency syndrome (AIDS) who was diagnosed with PICC-related bloodstream infection due to *T. pulmonis*.

## Case presentation

A 48-year-old Japanese man presented with symptoms of right-sided facial asymmetry and right hand palsy that had lasted for 10 days. He had a past medical history of syphilis, gonorrhea, varicella, hepatitis B viral infection, and chronic use of tobacco (1–2 packs of cigarettes per day for 28 years). Laboratory examinations revealed positivity for human immunodeficiency virus (HIV) antigen/antibody, a CD4 positive lymphocyte count of 84 cells/μL, and an HIV-1 RNA viral load of 180,000 copies/mL. Contrast-enhanced magnetic resonance imaging of the head showed three mass lesions in the brain, and the specimen biopsy from these lesions revealed diffuse large B cell lymphoma. He was diagnosed with AIDS-associated primary brain malignant lymphoma. The patient was administered emtricitabine/tenofovir disoproxil combination therapy and raltegravir as the antiretroviral therapy. Atovaquone and azithromycin were also used for prophylaxis of pneumocystis pneumonia and nontuberculous mycobacterial infections.

A PICC was implanted, and he received five courses of R-CHOP chemotherapy (rituximab, cyclophosphamide, doxorubicin hydrochloride, vincristine, and prednisone). Five days after the last R-CHOP chemotherapy course, he suddenly developed a fever with shivering chills. His white blood cell count was 4300/μL, and his neutrophil count was 4200/μL. The CD4-positive lymphocyte cell count was 228 cells/μL, and the HIV-1 RNA viral load was 20 copies/mL. We administered 2 g cefozopran hydrochloride, which is a fourth generation cephalosporin, and vancomycin on day 1 (every 12 h) and on day 3, respectively, and the PICC was removed. The patient’s condition improved and become afebrile within 48 h after the initiation of cefozopran hydrochloride. Cultures obtained from blood and revealed gram-positive rods, which were identified as *T. pulmonis* using 16S ribosomal RNA gene sequencing. The colony count of *T. pulmonis* grown from PICC culture was 10^3^ colony-forming units. Thus, he was diagnosed with *T. pulmonis* bacteremia resulting from a PICC-related bloodstream infection. Because of the results of the susceptibility test (Table 1), we continued cefozopran hydrochloride for 16 days. Follow up blood culture on 5 days after treatment was negative and clinical relapse was not observed. He remains in complete remission as of this report.

**Table 1:** Minimum inhibitory concentrations (MICs) of the *Tsukamurella pulmonis* strain in the present case

Antibiotics	MIC (mg/mL)	Antibiotics	MIC (mg/mL)
Penicillin G	8	Gentamicin	2
Ampicillin	≥16	Amikacin	≤4
Ceftriaxone	0.5	Minomycin	≤0.5
Cefozopran	1	Clindamycin	≥2
Cefepime	≤1	Vancomycin	2
Imipenem	≤1	Levofloxacin	≤0.25
Meropenem	0.5		

## Discussion and conclusions

Although *T. pulmonis* generally causes CLABSI from CVCs and Hickman catheters, we report a case of PICC-related bloodstream infection due to this microorganism and review the related literature.

Table 2 summarizes the seven reported cases of *T. pulmonis* bacteremia in the literature [2, 7, 11–14]. Three patients had severe immunodeficiency combined with bone marrow transplantation, IgA nephropathy, or metastatic breast cancer as the underlying disease [7]. Another three cases had hematological malignancy. One patient had an intracerebral hemorrhage and CLABSI [14]. CVCs or Hickman-catheters were implanted in all of these cases. Therefore, a relationship between CVCs or Hickman-catheters and the presence of *T. pulmonis* bacteremia exists; however, the risk of *T. pulmonis* bacteremia has not been reported with PICCs.

**Table 2:** Summary of case reports of *Tsukamurella pulmonis* bacteremia

Age (years)	Sex	Diseases	Catheter	Transplantation	Antibiotics	Outcome	Reference
1.5	M	SCID	Hickman	+	Amikacin, clarithromycin, ceftriaxone, vancomycin	Survived	[7]
3	F	ALL	CVC	–	Cefixime, ertapenem, ciprofloxacin, amikacin oral clarithromycin	Survived	[13]
38	F	High-grade Burkitt-like lymphoma	Hickman	+	Teicoplanin	Survived	[12]
39	M	ALL	Hickman	No data	Vancomycin, meropenem, amphotericin B	No data	[11]
48	F	IgA nephropathy	CVC	–	Ciprofloxacin, gentamicin, vancomycin	No data	[7]
48	M	Intracerebral hemorrhage	CVC	–	Ceftazidime, vancomycin	Survived	[14]
67	F	Metastatic breast cancer	CVC	–	Sulfamethoxazole-trimethoprim	No data	[7]

*SCID* severe combined immune deficiency, *ALL* acute lymphocytic leukemia, *CVC* central venous catheter

However, a recent systematic review and meta-analysis showed that the reduced risk of CLABSI with PICCs in comparison to CVCs was greatest for outpatients (RR, 0.22; 95% CI, 0.18–0.27) than for hospitalized patients (RR 0.73; 95% CI, 0.54–0.98) and PICC-associated CLABSI occurred as frequently as CVC-associated CLABSI in hospitalized patients (incidence rate ratio 0.91; 95% CI, 0.46–1.79) [15]. Thus, PICCs may also be a risk factor for CLABSI due to *T. pulmonis* bacteremia.

The appropriate treatment for *T. pulmonis* bacteremia remains unknown because standard susceptibility methods for this organism have not been established. There have been a few reports describing various treatments based on susceptibility methods (Table 2), including the E-test using Mueller-Hinton agar plates [13] and the disk diffusion technique [12]. In the present case, minimum inhibitory concentration (MIC) results using the broth dilution method showed susceptibility to vancomycin and cefozopran hydrochloride (Table 1). We continued cefozopran hydrochloride only because of the relatively high MIC for vancomycin. Although further studies are needed to confirm the standard susceptibility methods and optimal susceptibility interpretations, fourth generation cephalosporins including cefozopran hydrochloride and cefepime may be effective against *T. pulmonis* and may represent appropriate treatment for *T. pulmonis* bacteremia.

The relation between AIDS and the incidence of *T. pulmonis* bacteremia is unknown. *T. pulmonis* bacteremia has not been previously reported in AIDS patients, although an advanced-stage AIDS patient with *Tsukamurella* spp. pneumonia was reported by Alcaide et al. [16]. When the CD4-positive lymphocyte cell count is over 200 cells/μL, the defect in cell mediated immunity is mild to moderate, not severe, and it is not clear if this defect is sufficient to explain the predisposition to *Tsukamurella* infection.

In conclusion, we encountered a case of PICC-related bloodstream infection due to *T. pulmonis*. The present case suggests that PICCs may be a risk factor for developing *T. pulmonis* bacteremia, and fourth-generation cephalosporins may be effective treatment.

## Abbreviations

AIDS: Acquired Immunodeficiency Syndrome
ALL: Acute lymphocytic leukemia
CI: confidence interval
CLABSI: catheter-line associated bloodstream infection
DLBCL: diffuse large B cell lymphoma
HIV: human immunodeficiency virus
PICCs: peripherally inserted central catheters
RR: relative risk
SCID: severe combined immune deficiency
